# Oral Bicarbonate Therapy in Non-Haemodialysis Dependent Chronic Kidney Disease Patients: A Systematic Review and Meta-Analysis of Randomised Controlled Trials

**DOI:** 10.3390/jcm8020208

**Published:** 2019-02-07

**Authors:** May Khei Hu, Miles D. Witham, Roy L. Soiza

**Affiliations:** 1 NHS Grampian, Aberdeen Royal Infirmary, Foresterhill, Aberdeen AB25 2ZN, UK; roy.soiza@nhs.net; 2 NIHR Newcastle Biomedical Research Centre, Newcastle University and Newcastle-upon-Tyne Hospitals Trust, Newcastle upon Tyne NE4 5PL, UK; miles.witham@newcastle.ac.uk

**Keywords:** bicarbonate, acid-base balance, CKD

## Abstract

Metabolic acidosis is a common complication in chronic kidney disease (CKD) patients, and is associated with an accelerated decline in renal function. Oral bicarbonate therapy has been used to counteract metabolic acidosis in CKD for decades. However, until recently, there have been very few intervention studies testing the effectiveness of bicarbonate therapy at improving metabolic acidosis or its consequences in patients with CKD. In this systematic review and meta-analysis, we aimed to examine the outcomes of all published randomised controlled trials (RCTs) that investigated the effect of oral bicarbonate therapy in adults with CKD. Ovid MEDLINE^®^, EMBASE^®^ and Cochrane Library were searched in mid-October 2018 for English literature, with no restrictions applied to the publication status or date. Seven RCTs that recruited 815 participants met our inclusion criteria after full text review. Oral bicarbonate supplementation resulted in a slightly higher estimated glomerular filtration rate (eGFR) (mean difference 3.1 mL/min per 1.73 m^2^; 95% CI 1.3–4.9) and serum bicarbonate levels (mean difference 3.4 mmol/L; 95% CI 1.9–4.9) at the end of follow-up (three months to five years) compared to those given placebo or conventional CKD treatment. When limited to studies reporting outcomes at one year, the positive effect of oral bicarbonate therapy on eGFR was attenuated. There were no significant treatment effects in other parameters such as systolic blood pressure (BP) and weight. These findings should be interpreted with caution and further trial evidence is needed to establish the net overall benefit or harm of oral bicarbonate therapy in CKD.

## 1. Introduction

More than 1.8 million people in England are known to have chronic kidney disease (CKD), while another million of the population are thought to have the condition but are yet to be diagnosed [1,2]. CKD can significantly reduce quality and quantity of life and may lead to the need for renal replacement therapy. Around 45,000 premature deaths are attributed to CKD annually in the United Kingdom [3]. Furthermore, the management of CKD places a substantial economic burden on healthcare systems across the globe, and healthcare expenditure has been noted to be inversely proportional to renal function [4]. The National Health Service (NHS) in England spent approximately £1.45 billion on CKD in 2009–2010 [1]. Given the impact of CKD on quality of life and healthcare expenditure, interventions that may slow the progression of CKD are of great importance.

Metabolic acidosis is frequently found in CKD patients, and becomes more common with worsening renal function [4]. Metabolic acidosis has been operationalised as serum bicarbonate levels that are consistently below 22 mmol/L [4,5,6]. It is associated with other pathophysiological changes including systemic inflammation, upregulation of parathyroid hormone receptors in osteoblasts and increased activity of osteoclasts; which leads to accelerated bone resorption and osteopenia, reduction of Na^+^-K^+^-ATPase activity in erythrocytes—resulting in impairment of myocardial contractility and heart failure, and reduced respiratory reserve and exhaustion of body buffer systems, which increases severity of acute intercurrent illnesses [4,5,6,7,8,9,10]. The degree of metabolic acidosis is directly associated with glomerular filtration rate GFR, and is due to the failure of the kidneys to produce ammonia, regenerate bicarbonate and excrete hydrogen ions [11]. Importantly, metabolic acidosis is associated with an accelerated decline in renal function, an association that may be causal in nature [12].

As part of CKD patients’ care, oral alkali such as bicarbonates and citrates, base-producing fruits and vegetables or low protein diets are commonly prescribed to avoid or correct metabolic acidosis. Oral bicarbonate therapy has been used to counteract metabolic acidosis in CKD for decades. However, until recently, there have been very few intervention studies testing the effectiveness of bicarbonate therapy at improving metabolic acidosis, or its consequences in patients with CKD, and there are no systematic reviews evaluating the effectiveness and safety of this approach. This is reflected by the current guidelines, which are still unable to make an evidence-based recommendation regarding the correction of mild-to-moderate acidosis in CKD [13]. To date, there is also no consensus on the optimal range for serum bicarbonate in CKD patients. The paucity of clear guidelines results in variability in clinical practice when prescribing oral bicarbonate therapy for patients with CKD and metabolic acidosis [13].

In this systematic review and meta-analysis, we aimed to examine the outcomes of all published randomised controlled trials (RCTs) that investigated the effect of oral bicarbonate therapy in non-haemodialysis dependent adults with CKD.

## 2. Methods

### 2.1. Literature Search Strategy

The review protocol was registered in the PROSPERO database (Ref: CRD42018112908) [14]. Three databases (Ovid MEDLINE^®^, EMBASE^®^ and Cochrane Library) were searched in mid-October 2018 for English literature, with no restrictions applied to the publication status or date. Grey literature was not searched. The full search strategy is presented in Appendix A.

### 2.2. Inclusion and Exclusion Criteria

Our inclusion criteria were randomised controlled trials, availability of full text in English language, all aetiologies and severities of CKD, comparison of bicarbonate therapy with placebo or standard care, and any health-related outcome measures. Our exclusion criteria were children under the age of 18, people receiving haemodialysis and the comparison of bicarbonate therapy with active treatment.

### 2.3. Study Selection

Two reviewers independently screened all titles and abstracts. The bibliographies of selected articles were hand-searched for any other potentially relevant studies. Any uncertainties about study eligibility were discussed between reviewers, and if necessary, with a third reviewer.

### 2.4. Data Extraction

A data extraction form was designed by adapting and customising the Cochrane collaboration’s data collection form for intervention review—RCTs and non-RCTs [15]. Two independent assessors performed the data extraction. Data discrepancies were resolved by discussion and consensus between the two assessors.

### 2.5. Risk of Bias

Risk of bias was evaluated using the Cochrane collaboration’s risk of bias tool [15]. Criteria assessed included random sequence generation, allocation concealment, blinding of participants and personnel, blinding of outcome assessment, incomplete outcome data, selective reporting and other biases. Risk was reported as low, high or unclear. Results from the assessment were subsequently tabulated using RevMan 5.3 (The Cochrane Collaboration, Copenhagen, Denmark) to generate a risk of bias graphic and summary table [16].

### 2.6. Outcome Measures and Data Synthesis

The primary outcome measure of interest was the rate of change in estimated glomerular filtration rate (eGFR). Secondary outcome measures were eGFR at the end of follow-up, mortality, blood pressure, number of patients proceeding to renal replacement therapy and quality of life. Meta-analyses of these and any other health-related outcome measures were performed when there were at least three trials reporting the same outcome measure. The random effects model was used for continuous data and forest plots generated using RevMan 5.3. Statistical heterogeneity in treatment effects was determined using the *I*^2^ test.

### 2.7. Subgroup Analysis

Subgroup analyses were planned by mean study age, mean proportion of men and the duration of the study.

## 3. Results

### 3.1. Study Selection

**Figure 1:**
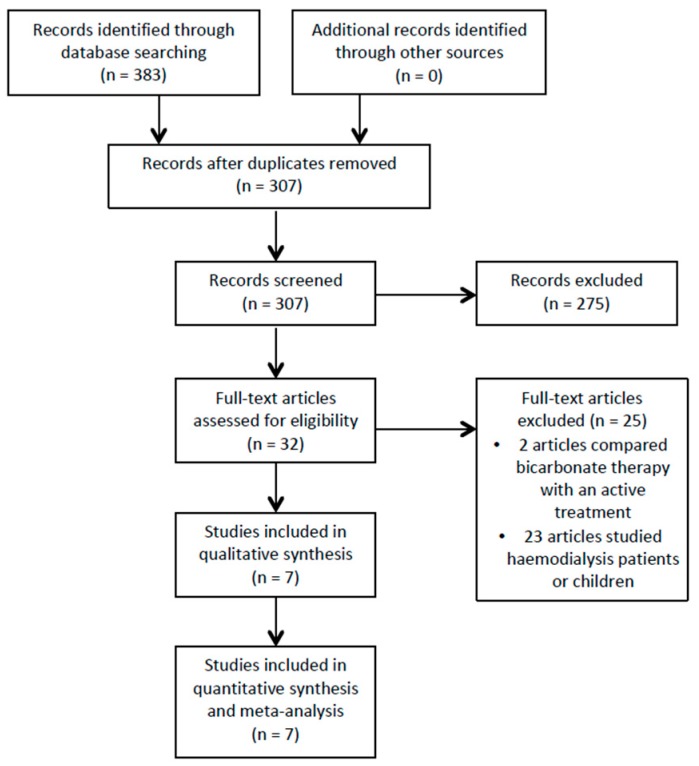
Study flow diagram.

After deduplication, the search identified 307 potentially relevant articles, and 32 trials were shortlisted after reading titles and abstracts (see Figure 1). Seven trials that recruited 815 participants met the inclusion criteria after a full text review (see Table 1). Two trials were set in the USA and India, respectively, with one trial each from Italy, South Korea and the UK.

### 3.2. Risk of Bias Analysis

**Figure 2:**
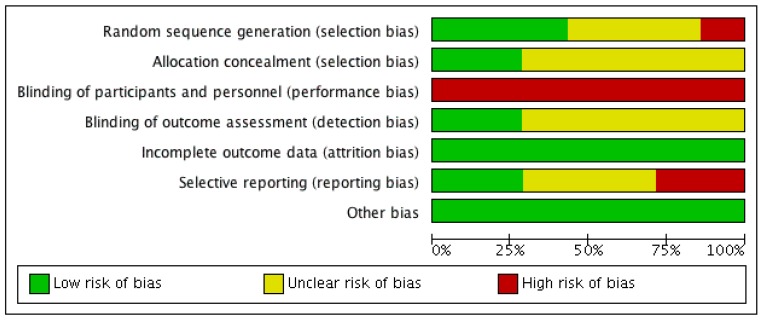
Risk of bias graph (presented as percentages across all included studies) illustrating the review authors’ judgements about each risk of bias criterion.

**Figure 3:**
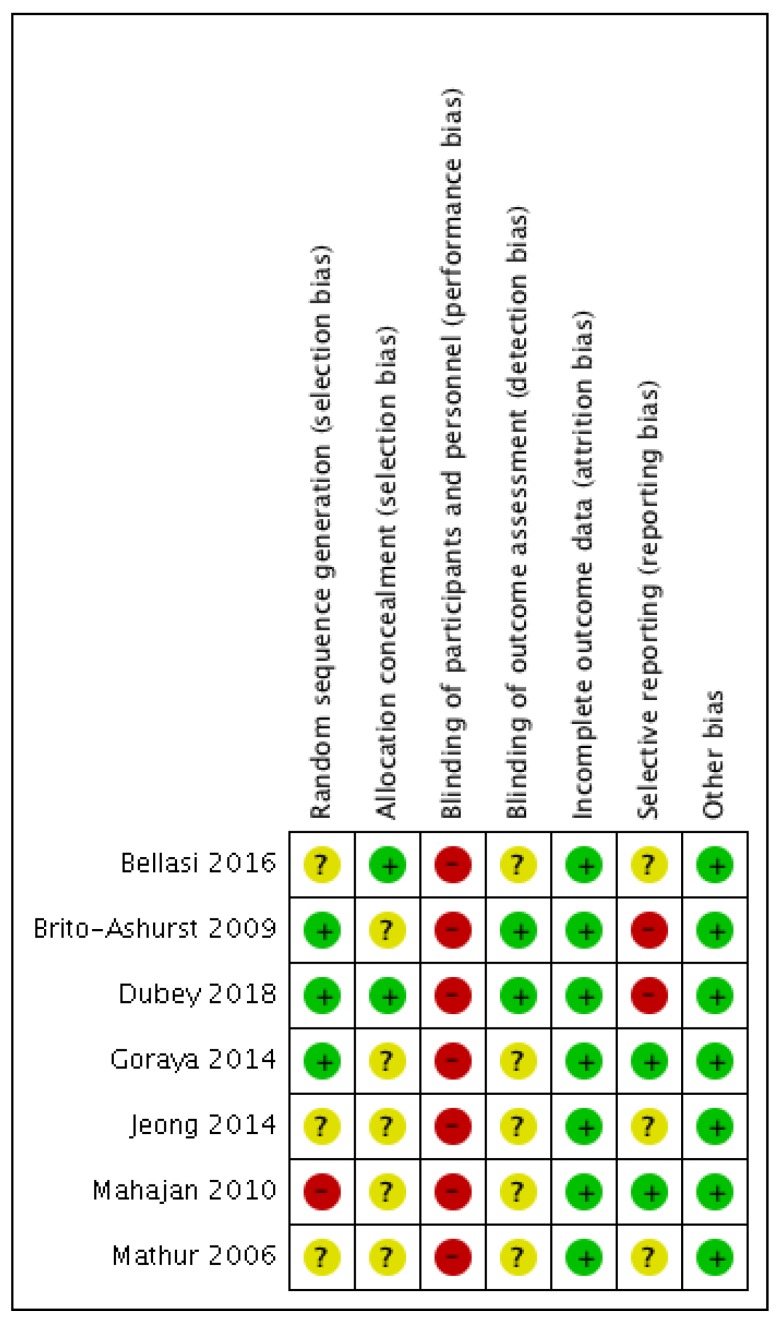
Risk of bias table illustrating the review authors’ judgements about each risk of bias criterion. Green: Low risk of bias; Yellow: Unclear risk of bias; Red: High risk of bias.

Three RCTs described utilisation of adequate randomisation processes [18,21,22] to minimise selection bias, while one study used patient’s identifying number [19], which was likely to have increased the risk of bias (see Figure 2 and Figure 3). Two RCTs applied allocation concealment by employing a central randomisation process [22] or using opaque sequenced envelopes [23], but there was no mention of allocation concealment in the other studies. All studies had a high risk of performance bias due to the nature of the intervention, in which oral bicarbonate therapy was titrated and monitored to achieve desired serum bicarbonate levels. Two studies used placebo as a comparator [17,19], which might potentially reduce the risk of performance bias, but did not completely abolish it.

Two RCTs reported blinding of their outcome assessors [18,23] while it was unclear in the rest if this risk of bias was minimised or eliminated. All studies were deemed to have low attrition bias as the dropout rate was less than 10% and accounted for in each study. The missing outcomes were also thought to be insufficient to have a clinically relevant impact on the observed effect size. Two studies recorded all expected outcomes using measurements and analysis methods that were pre-specified [19,21], and therefore had a low reporting bias. Two RCTs were judged to have a high risk of bias because outcome measures were detailed with an unconventional outcome measure (decline in creatinine clearance) [18], and measurements (mean and 95% confidence interval (CI)) [23]. The remaining studies did not offer sufficient information to permit judgement about this criterion.

### 3.3. Outcomes

#### 3.3.1. Serum Bicarbonate

**Figure 4:**
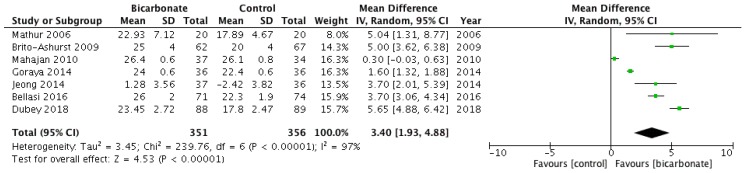
Forest plot comparing the effects of oral bicarbonate therapy and control on serum bicarbonate levels. Note: Boxes represent the mean difference between bicarbonate and control in individual trials. The boxes are proportional to the weight of each study in the analysis and the lines represent their 95% confidence interval (CI). The diamond represents the pooled mean difference, and its width represents its 95% CI. SD: Standard deviation; CI: Confidence interval; Tau^2^: Variance of the effect size across studies; Chi^2^: Weighted sum of squared differences between individual studies and the pooled effect across studies; df: Degrees of freedom; *I*^2^: Percentage of variation across studies that is due to heterogeneity; Z: Test for overall effect across all studies.

All seven RCTs studied serum bicarbonate levels following the randomisation of study participants (see Figure 4) [17,18,19,20,21,22,23]. Data from 707 patients were analysed; 57.9% were males and their mean age ranged from 37.5 ± 17 years to 65.5 ± 11.4 years. Serum bicarbonate levels were higher after oral bicarbonate therapy (mean difference 3.4 mmol/L; 95% CI 1.9–4.9) but the results were highly heterogeneous (*I*^2^ = 97%).

#### 3.3.2. eGFR and Serum Creatinine

**Figure 5:**
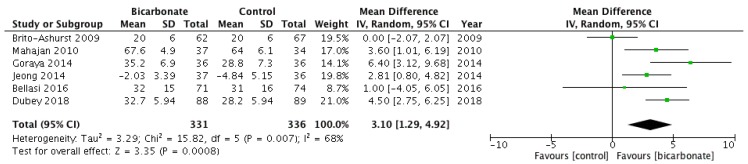
Forest plots comparing the effects of oral bicarbonate therapy and control on eGFR and serum creatinine levels. Note: Boxes represent the mean difference between bicarbonate and control in individual trials. The boxes are proportional to the weight of each study in the analysis and the lines represent their 95% confidence interval (CI). The diamond represents the pooled mean difference, and its width represents its 95% CI. SD: Standard deviation; CI: Confidence interval; Tau^2^: Variance of the effect size across studies; Chi^2^: Weighted sum of squared differences between individual studies and the pooled effect across studies; df: Degrees of freedom; *I*^2^: Percentage of variation across studies that is due to heterogeneity; Z: Test for overall effect across all studies.

Six RCTs [18,19,20,21,22,23] investigated eGFR after randomising patients to oral bicarbonate therapy or placebo (see Figure 5). Data were not presented in a format that allowed the rate of change of eGFR (the primary outcome measure) to be analysed, so eGFR at the end of follow-up was used. A total of 667 patients were analysed; 57.7% were males and their mean age ranged from 50.1 ± 11.6 years to 65.5 ± 11.4 years. eGFR favoured bicarbonate therapy (mean difference 3.1 mL/min per 1.73 m^2^; 95% CI 1.3–4.9) but the analysis revealed moderately high heterogeneity (*I*^2^ = 68%).

Four studies measured serum creatinine at the end of their follow-up period (see Supplementary Figure S1) [17,19,20,23]. Data from 361 patients were included; 61% were males and their mean age ranged from 37.5 ± 17 years to 55.8 ± 12.7 years. Compared with placebo and standard care for CKD, oral bicarbonate supplementation had non-significant effects on serum creatinine (*p* = 0.09).

#### 3.3.3. Systolic Blood Pressure

**Figure 6:**
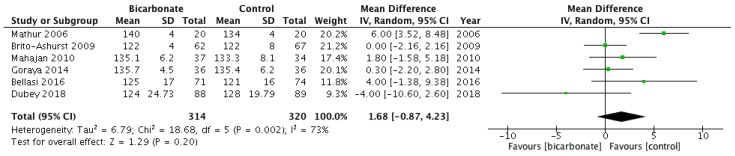
Forest plot comparing the effects of oral bicarbonate therapy and control on systolic blood pressure. Note: Boxes represent the mean difference between bicarbonate and control in individual trials. The boxes are proportional to the weight of each study in the analysis and the lines represent their 95% confidence interval (CI). The diamond represents the pooled mean difference, and its width represents its 95% CI. SD: Standard deviation; CI: Confidence interval; Tau^2^: Variance of the effect size across studies; Chi^2^: Weighted sum of squared differences between individual studies and the pooled effect across studies; df: Degrees of freedom; *I*^2^: Percentage of variation across studies that is due to heterogeneity; Z: Test for overall effect across all studies.

Six studies recorded systolic blood pressure (BP) as an outcome measure (see Figure 6) [17,18,19,21,22,23]. A total of 635 patients were included; 57.3% were males and their mean age ranged from 37.5 ± 17 years to 65.5 ± 11.4 years. Oral bicarbonate therapy had uncertain effects on systolic BP when compared to placebo or conventional treatment for CKD (*p* = 0.19).

#### 3.3.4. Weight

**Figure 7:**
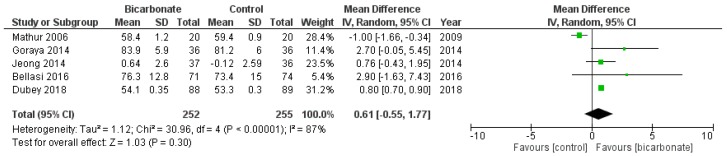
Forest plot comparing the effects of oral bicarbonate therapy and control on weight. Note: Boxes represent the mean difference between bicarbonate and control in individual trials. The boxes are proportional to the weight of each study in the analysis and the lines represent their 95% confidence interval (CI). The diamond represents the pooled mean difference, and its width represents its 95% CI. SD: Standard deviation; CI: Confidence interval; Tau^2^: Variance of the effect size across studies; Chi^2^: Weighted sum of squared differences between individual studies and the pooled effect across studies; df: Degrees of freedom; *I*^2^: Percentage of variation across studies that is due to heterogeneity; Z: Test for overall effect across all studies.

Five RCTs reported the weight of their patients at the conclusion of their studies (see Figure 7) [17,20,21,22,23]. 507 patients were analysed; 69.3% were males and their mean age ranged from 37.5 ± 17 years to 65.5 ± 11.4 years. The effects of oral bicarbonate therapy on weight of CKD patients are uncertain (*p* = 0.3) and the results are highly heterogeneous (*I*^2^ = 87%).

#### 3.3.5. Other Outcomes

The rate of change of eGFR was intended as a primary outcome, but data from multiple trials were not presented in a format that enabled this parameter to be analysed, so eGFR at the end of follow-up was used. Other listed outcomes that were not available included mortality rate, number of patients proceeding to renal replacement therapy and quality of life.

### 3.4. Subgroup Analyses

Subgroup analyses were performed by the duration of the study, thereby eliminating one source of heterogeneity. These analyses were only possible for eGFR and serum bicarbonate at one year follow-up (see Supplementary Figures S2 and S3), as there were too few studies or insufficient data in other outcome measures to allow for meaningful analysis.

Three studies investigated eGFR and serum bicarbonate levels at one year after the randomisation of patients to oral bicarbonate therapy or placebo treatment [18,20,22]. Of the 347 patients that were analysed; 55.4% were males and their mean age ranged from 53.3 ± 13.5 years to 65.5 ± 11.4 years. The effects of bicarbonate therapy on eGFR were non-significant when the duration of the study was standardised at one year (*p* = 0.19). The heterogeneity of treatment results was lower than other analyses (*I*^2^ = 46%). Serum bicarbonate at one year was higher after oral bicarbonate therapy (mean difference 3.2 mmol/L, 95% CI 2.0–4.3), but there was still significant heterogeneity (*I*^2^ = 66%).

## 4. Discussion

### 4.1. Outcomes

Oral bicarbonate supplementation resulted in a slightly higher eGFR at the end of follow up (three months to five years) compared to those given placebo or conventional CKD treatment. Bicarbonate therapy also improved serum bicarbonate levels by an average of 3.2 mmol/L compared to the control arm, but we did not find any significant treatment effects in other parameters such as systolic BP and weight.

These findings should be interpreted with caution due to a high level of heterogeneity between studies. The heterogeneity probably reflects the marked differences across trials, ranging from population demographics to dosing regimen and length of follow-up. Furthermore, the mean age of trial participants was remarkably low despite the increased prevalence of CKD in older people [1,2]. All but one trial recruited patients with a mean age below 56 years. Although most epidemiological studies observed that CKD was more common in women, all seven RCTs in this review enrolled more men than women [24]. This suggests that there may be a degree of selection bias in trial recruitment that may limit the generalisation of results in clinical practice. Some trials recruited atypical populations, e.g., those with stage 2 CKD with albuminuria due to hypertension [19] or those with CKD of unknown aetiology in India [17]. It is not clear that the observed treatment effects would be seen in more typical patients with CKD. Patients’ dietary intakes were not clearly accounted for, and this might have influenced the serum bicarbonate levels, as acid- and base-producing diets are known to skew bicarbonate levels. Patients also had various co-morbidities such as cardiovascular diseases and type 2 diabetes mellitus, but this confounding factor might be difficult to avoid when recruiting CKD patients, as most patients have at least one, if not more, co-morbidities.

Almost all trials included in this analysis were open-label, and it is known that unmasked trial designs tend to overestimate the treatment effect size compared to placebo. Several trials adopted a ‘treat to target’ approach for bicarbonate, in which doses were escalated in the intervention arm to try and reach a pre-defined bicarbonate level. Such approaches again tend to magnify treatment effects compared to the comparison of bicarbonate at a fixed dose with controls, although it can be argued that such an approach better approximates clinical practice.

Another likely confounding factor is the duration of study. When limited to studies reporting outcomes at one year of follow-up, the positive effect of oral bicarbonate therapy on eGFR was attenuated and eGFR did not differ significantly between those on bicarbonate supplementation and placebo. All trials also recruited patients with a wide range of baseline bicarbonate levels (16 to 24 mmol/L), and this may have had an influence on the observed response to treatment, such that studies that began with lower baseline bicarbonate levels might have witnessed a larger treatment effect size.

Our results revealed that a modest total of 815 participants worldwide had been recruited into seven RCTs of bicarbonate therapy. It is highly likely that future studies will influence the results of the meta-analysis. The authors are aware of four further relevant RCTs that have published their protocols but are yet to report their results—BiCARB [13], UBI [25], SoBic [26] and BASE [27]. Their planned recruitment figures should more than double the number of participants in trials of bicarbonate treatment in CKD to date, as well as address current weaknesses in the evidence base, such as a lack of older people and data on physical function and quality of life.

### 4.2. Potential Adverse Effects of Bicarbonate Oral Therapy

As with most medications, oral bicarbonate therapy comes with its own adverse effects and cautions. Sodium bicarbonate tablets are awkward to take, especially for older people with impaired swallowing and polypharmacy; the tablets are large and several tablets are usually required to be taken at one time or in a day [13]. The British National Formulary (BNF) listed abdominal discomfort and bloating as recognised side effects, which may lead to compliance issues in patients [13]. 600 mg sodium bicarbonate tablets taken three times daily cost the NHS £190 per patient per year, despite a lack of robust evidence supporting the efficacy of this treatment.

Sodium bicarbonate tablets also contain 6 mmol of sodium in every 500 mg, which could contribute to hypertension and fluid overload if not monitored closely, especially in a population with impaired renal clearance [24]. This finding is corroborated by observational studies, which suggested that elevated serum bicarbonate levels were associated with an increased risk of heart failure in CKD patients [28]. It is therefore reassuring that this meta-analysis did not demonstrate any worsening of systolic blood pressure with bicarbonate therapy.

The targeted serum bicarbonate level, dose and time of initiation are yet to be determined. Despite new evidence suggesting the range of potential benefits, overtreatment with oral alkali therapy may result in metabolic alkalosis, which is also associated with poor outcomes in patients with CKD [11]. An alkaline pH has also been shown to augment vascular calcification in animal models [11]. As arterial elasticity declines with age and older people are more likely to have CKD, caution has to be exercised when considering oral bicarbonate therapy.

### 4.3. Strengths and Limitations

To our knowledge, this is the first synthesis of trials investigating the effects of bicarbonate therapy. All fully published trials were included and we undertook meta-analysis where possible. However, there was substantial heterogeneity in all included studies. The seven RCTs had important differences in various parameters including population demographics, intervention regimes, outcome measures and duration of study. Additionally, studies were often at high risk of bias but poorer quality studies could not be excluded due to the limited number of published RCTs that suited our inclusion criteria.

## 5. Conclusions

Bicarbonate supplementation modestly improved eGFR and serum bicarbonate levels compared to placebo or conventional CKD management. Evidence of improvement in other health-related outcome measures was lacking. These findings should be interpreted with caution due to high heterogeneity and risk of bias in studies. Further trial evidence is needed to establish the net overall benefit or harm of oral bicarbonate therapy in CKD, and to define the target groups most likely to benefit from treatment.

## Figures and Tables

**Table 1:** Characteristics of included randomised controlled trial.

Study	Location	*n*	Mean Age (Years)	CKD Stage	Bicarbonate Level Entry Criterion	Intervention	Comparator	Duration	Primary Outcome
Mathur et al., 2006 [17]	India	40	41	“Mild to moderate” CKD (creatinine < 442 μmol/L). CKD stage not specified	Not specified	Oral bicarbonate 1.2 mEq/kg in 3 divided doses, titrated to maintain serum bicarbonate in range 22–26 mmol/L	Placebo	3 months	Not specified
De Brito-Ashurt et al., 2009 [18]	UK	134	55	4 or 5	16 mmol/L < Bicarbonate < 19 mmol/L	Oral bicarbonate 600 mg 3×/day, increased as needed to maintain serum bicarbonate > 23 mmol/L	Usual care	2 years	Decline in creatinine clearance of >3 mL/min/year
Mahajan et al., 2010 [19]	USA	120	51	2 with hypertension and microalbuminuria	Total CO_2_ > 24.5 mmol/L	Oral bicarbonate 0.5 mEq/kg lean body weight	Placebo	5 years	eGFR decline rate
Jeong et al., 2014 [20]	South Korea	80	55	4 or 5	Total CO_2_ < 22 mmol/L	Oral bicarbonate 1 g 3×/day, titrated to maintain serum bicarbonate > 22 mmol/L	Usual care	12 months	eGFR
Goraya et al., 2014 [21]	USA	108	54	3	22 mmol/L < Total CO_2_ < 24 mmol/L	Oral bicarbonate 0.3 mEq/Kg lean body weight in three divided doses	Usual care	3 years	eGFR
Bellasi et al., 2016 [22]	Italy	145	65	3b or 4; in patients with T2DM	Bicarbonate < 24mmol/L	Oral bicarbonate 0.5 mEq/kg twice daily, until serum bicarbonate 24–28 mmol/L	Usual care	12 months	Insulin resistance
Dubey et al., 2018 [23]	India	188	50	3 and 4	Bicarbonate < 22mmol/L	Oral bicarbonate titrated with weekly monitoring	Usual care	6 months	Mid-arm muscle circumference

CKD: Chronic kidney disease; eGFR: Estimated glomerular filtration rate; CO_2_: Carbon dioxide; T2DM: Type 2 diabetes mellitus.

## Supplementary Materials

The following are available online at http://www.mdpi.com/2077-0383/8/2/208/s1, Supplementary Figure S1. Forest plot comparing the effects of oral bicarbonate therapy and control on serum creatinine levels. Supplementary Figure S2. Forest plot comparing the effects of oral bicarbonate therapy and control on eGFR at one year. Supplementary Figure S3. Forest plot comparing the effects of oral bicarbonate therapy and control on serum bicarbonate levels at one year.

## Appendix A. Search Strategy

**Table A1:** Search strategy in MEDLINE.

1	exp Bicarbonates [MeSH] or exp Sodium Bicarbonate [MeSH] or bicarbonates [tiab]	24,367
2	Chronic Kidney Disease or CKD [mp] or exp Renal Insufficiency, Chronic [MESH] or or Chronic Renal Insufficiency [tiab]	127,737
3	(exp Bicarbonates [MeSH] or exp Sodium Bicarbonate [MESH] or bicarbonates [tiab]) and (Chronic Kidney Disease or CKD [mp] or exp Renal Insufficiency, Chronic [MESH] or or Chronic Renal Insufficiency [tiab])	812
4	Limit 3 to RCT and English language	76

exp: Explode; MeSH: Medical Subject Headings; tiab: Title or abstract; mp: Keyword; RCT: Randomised controlled trials.

**Table A2:** Search strategy in EMBASE.

1	exp Bicarbonate [MeSH] or Bicarbonate.mp	69,535
2	exp Chronic Kidney Failure [MeSH] or (chronic kidney disease or chronic kidney failure or chronic kidney insufficiency or chronic renal disease or chronic renal failure or chronic renal insufficiency).mp	116,647
3	(exp Bicarbonate [MeSH] or Bicarbonate.mp) and (exp Chronic Kidney Failure [MeSH] or (chronic kidney disease or chronic kidney failure or chronic kidney insufficiency or chronic renal disease or chronic renal failure or chronic renal insufficiency).mp)	2323
4	Limit 3 to English language and exclude Medline journals and RCT	3

exp: Explode; MeSH: Medical Subject Headings; mp: Keyword; RCT: Randomised controlled trials.

**Table A3:** Search strategy in Cochrane.

1	MeSH descriptor. [Bicarbonates] explode all trees	1201
2	MeSH descriptor: [Sodium Bicarbonate] explode all trees	610
3	#1 or #2 or (Bicarbonate *)	3123
4	MeSH descriptor: [Renal Insufficiency, Chronic] explode all trees	5705
5	Chronic kidney disease or chronic kidney failure or chronic kidney insufficiency or chronic renal disease or chronic renal failure or chronic renal insufficiency	13,858
6	#4 or #5	13,524
7	#3 and #6 in Trials	304

MeSH: Medical Subject Headings; * (truncation symbol) is used to search for multiple variants of a word all at once.
