# Supplementary material for: Oral Bicarbonate Therapy in Non-Haemodialysis Dependent Chronic Kidney Disease Patients: A Systematic Review and Meta-Analysis of Randomised Controlled Trials
Source: J Clin Med. 2019 Feb 7;8(2):208. doi: 10.3390/jcm8020208 (PMC6406285; doi:10.3390/jcm8020208)
Supplement: Supplementary file 1 [file jcm-08-00208-s001.pdf]

Supplementary Material

Study or Subgroup	Bicarbonate Mean	Bicarbonate SD	Bicarbonate Total	Control Mean	Control SD	Control Total	Weight	Mean Difference IV, Random, 95% CI	Year
Mathur 2006	2.86	1.21	20	3.62	1.18	20	7.7%	−0.76 [−1.50, −0.02]	2006
Mahajan 2010	1.06	0.05	37	1.12	0.1	34	47.9%	−0.06 [−0.10, −0.02]	2010
Jeong 2014	1.4	1.73	37	1.4	1.47	36	7.8%	0.00 [−0.74, 0.74]	2014
Dubey 2018	2.3	0.74	88	2.6	0.49	89	36.5%	−0.30 [−0.49, −0.11]	2018
**Total (95% CI)**			**182**			**179**	**100.0%**	**−0.20 [−0.42, 0.03]**	

Heterogeneity: Tau² = 0.03; Chi² = 9.57, df = 3 (P = 0.02); I² = 69%
Test for overall effect: Z = 1.72 (P = 0.09)

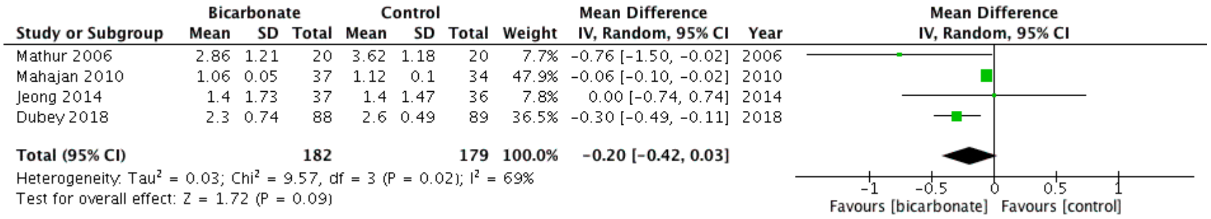

Supplementary Figure S1. Forest plot comparing the effects of oral bicarbonate therapy and control on serum creatinine levels.

Study or Subgroup	Bicarbonate Mean	Bicarbonate SD	Bicarbonate Total	Control Mean	Control SD	Control Total	Weight	Mean Difference IV, Random, 95% CI	Year
Brito-Ashurst 2009	20	6	62	20	6	67	42.6%	0.00 [−2.07, 2.07]	2009
Jeong 2014	−2.03	3.39	37	−4.84	5.15	36	43.8%	2.81 [0.80, 4.82]	2014
Bellasi 2016	32	15	71	31	16	74	13.6%	1.00 [−4.05, 6.05]	2016
**Total (95% CI)**			**170**			**177**	**100.0%**	**1.37 [−0.69, 3.43]**	

Heterogeneity: Tau² = 1.48; Chi² = 3.68, df = 2 (P = 0.16); I² = 46%
Test for overall effect: Z = 1.30 (P = 0.19)

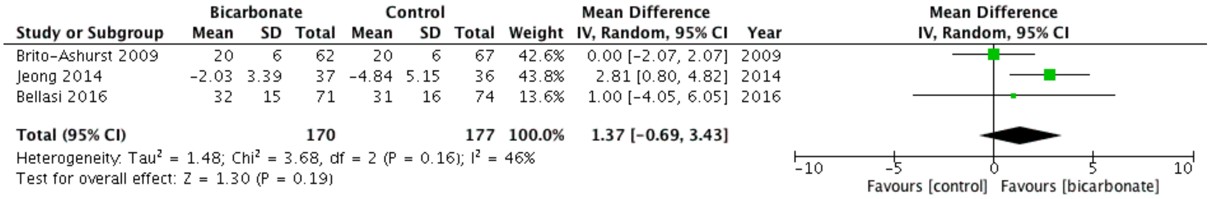

Supplementary Figure S2. Forest plot comparing the effects of oral bicarbonate therapy and control on eGFR at one year.

Study or Subgroup	Bicarbonate Mean	Bicarbonate SD	Bicarbonate Total	Control Mean	Control SD	Control Total	Weight	Mean Difference IV, Random, 95% CI	Year
Brito-Ashurst 2009	22	4	62	20	3	67	32.0%	2.00 [0.77, 3.23]	2009
Jeong 2014	1.28	3.56	37	−2.42	3.82	36	23.9%	3.70 [2.01, 5.39]	2014
Bellasi 2016	26	2	71	22.3	1.9	74	44.1%	3.70 [3.06, 4.34]	2016
**Total (95% CI)**			**170**			**177**	**100.0%**	**3.16 [2.02, 4.29]**	

Heterogeneity: Tau² = 0.66; Chi² = 5.96, df = 2 (P = 0.05); I² = 66%
Test for overall effect: Z = 5.44 (P < 0.00001)

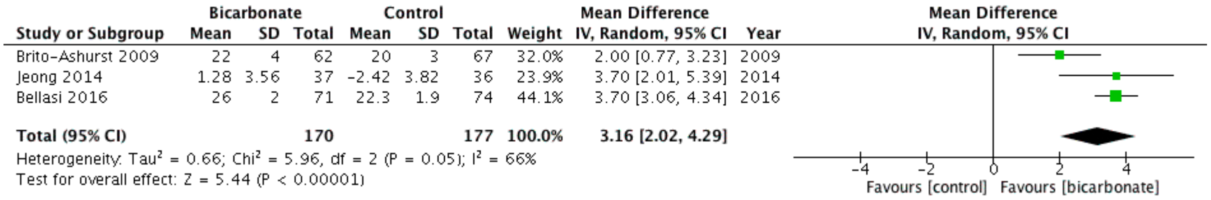

Supplementary Figure S3. Forest plot comparing the effects of oral bicarbonate therapy and control on serum bicarbonate levels at one year.
